# Automated Detection of Cancer Associated Genes Using a Combined Fuzzy-Rough-Set-Based F-Information and Water Swirl Algorithm of Human Gene Expression Data

**DOI:** 10.1371/journal.pone.0167504

**Published:** 2016-12-09

**Authors:** Pugalendhi Ganesh Kumar, Muthu Subash Kavitha, Byeong-Cheol Ahn

**Affiliations:** 1 Department of Information Technology, Anna University Regional Campus, Coimbatore, India; 2 Department of Computer Vision and Image Processing, School of Electronics Engineering, Kyungpook National University, Daegu, South Korea; 3 Department of Nuclear Medicine, Kyungpook National University School of Medicine and Hospital, Daegu, South Korea; Southwest University, CHINA

## Abstract

This study describes a novel approach to reducing the challenges of highly nonlinear multiclass gene expression values for cancer diagnosis. To build a fruitful system for cancer diagnosis, in this study, we introduced two levels of gene selection such as filtering and embedding for selection of potential genes and the most relevant genes associated with cancer, respectively. The filter procedure was implemented by developing a fuzzy rough set (FR)-based method for redefining the criterion function of f-information (FI) to identify the potential genes without discretizing the continuous gene expression values. The embedded procedure is implemented by means of a water swirl algorithm (WSA), which attempts to optimize the rule set and membership function required to classify samples using a fuzzy-rule-based multiclassification system (FRBMS). Two novel update equations are proposed in WSA, which have better exploration and exploitation abilities while designing a self-learning FRBMS. The efficiency of our new approach was evaluated on 13 multicategory and 9 binary datasets of cancer gene expression. Additionally, the performance of the proposed FRFI-WSA method in designing an FRBMS was compared with existing methods for gene selection and optimization such as genetic algorithm (GA), particle swarm optimization (PSO), and artificial bee colony algorithm (ABC) on all the datasets. In the global cancer map with repeated measurements (GCM_RM) dataset, the FRFI-WSA showed the smallest number of 16 most relevant genes associated with cancer using a minimal number of 26 compact rules with the highest classification accuracy (96.45%). In addition, the statistical validation used in this study revealed that the biological relevance of the most relevant genes associated with cancer and their linguistics detected by the proposed FRFI-WSA approach are better than those in the other methods. The simple interpretable rules with most relevant genes and effectively classified samples suggest that the proposed FRFI-WSA approach is reliable for classification of an individual’s cancer gene expression data with high precision and therefore it could be helpful for clinicians as a clinical decision support system.

## Introduction

Multiclass classification of gene expression data with a reduced number of genes remain challenging problems in cancer diagnosis. Microarrays and next-generation sequencing [1, 2] are the chief tools of cancer research for quantification of gene expression, DNA copy number, and microRNA activity of each individual. Hence, analyzing such data could give researchers useful information not only about the mechanism and cause of cancer but also a way to predict and prevent cancer and to find possible novel treatments. However, classification of multiclass data is more complex than binary data and further the classification accuracy may decline as the number of classes increased [3]. The implementation of artificial intelligence using data-mining tasks such as classification and clustering techniques has been applied to analyze gene expression values for cancer diagnosis [4–7]. However these techniques suffered by a greater computational cost and training time.

Rule-based approaches produced knowledge out of gene expression data with acceptable classification accuracy for diagnosing cancer. [8–11]. In addition some of the other approaches such as a decision tree [12] and ensemble classification tree [13, 14] have been used for identification of cancer-causing genes in gene expression data. Nonetheless, these approaches failed to consider the overlapping behavior of gene expression levels in uncertain situations. Data-driven approaches [15, 16] have been applied for extracting knowledge from the gene expression data without a human expert, but they were found to be weak in terms of the self-learning process. In general, these approaches are problematic for subtyping of cancer with identical expression levels in multiclass cancer data [17, 18].

In several studies the concept of fuzzy logic has been used to develop a rule-based system with the help of a learning algorithm to address multiclass issues among cancer genes as well as for suitable generation of if-then rules and a membership function (MF) for classification of a disease [19–25]. The genetic algorithm [20] and particle swarm optimization (PSO) techniques [21] can generate rules through simultaneous tuning of the MF, but it becomes too lengthy with more linguistic terms and was found to be incomprehensible for making diagnostic decisions. The ant bee algorithm [22] was recommended to produce compact if-then rules with better readability, but it results in consumption of more computation time because of the more complicated operations and more tunable control parameters. Fuzzy ontology [23] can extract the knowledge quickly, but its performance degrades with the scarce data distribution found in the multiclass gene expression data. The framework described in reference [24] transforms crisp rules into fuzzy rules using a stochastic global optimization procedure; however, the generation of the crisp rule using experts for multicategories of cancers is again a difficult task. Majority voting and fuzzy aggregation [25] are used in a multi-classification system, and it was reported that the combination of results from the individual classifiers for the final decision yields poor performance with more skewedness for the multiclass data on cancer gene expression.

Recently, fuzzy-rule-based multiclassification systems (FRBMS) [26] using combinations of methods were proposed, to take advantage of the crucial benefit of interpretability offered by the fuzzy system. Nevertheless, the presence of numerous genomic variables versus a relatively small number of patients poses challenges in understanding the data. Attempts have been made to use a genetic algorithm (GA) [27] in an FRBMS to perform classifier fusion and selection; this approach does not fulfill the skewness of the gene expression data. Furthermore, underfitting should be avoided during multiclassification because it results in a non-optimally robust system due to inadequate experimentation. To build a beneficial system for cancer diagnosis to overcome many shortcomings [28, 29] such as scarceness and highly nonlinear multicategory values, it is necessary to design an ideal method with precise principles of data analysis.

The abundance of genes expressed in microarray experiments requires a long computation time and results in complex output for an FRBMS. To implement an FRBMS for a gene expression-based cancer diagnosis problem, identification of most relevant genes associated with cancer from the large set of genes is mandatory [4, 15]. The purpose of this newly proposed combined fuzzy-rough-set-based f-information & water swirl algorithm (FRFI-WSA) approach was to design an FRBMS for analyzing gene expression data for cancer diagnosis. For an effective cancer diagnostic system, two levels of gene selection (by filtering and embedding procedures using 22 cancer gene expression datasets collected from various sources) were introduced. Next, we conducted a comparison of the performance of the proposed FRFI-WSA with GA, PSO, and artificial bee colony algorithm (ABC) for cancer gene expression datasets.

## Materials and Methods

### Cancer gene expression datasets

This study includes 22 gene expression datasets including name, number of genes (#Genes), samples (#Sam), and categories (#Cat) along with the source of collection and its type (Table 1). The performance of the proposed algorithm for classifying datasets irrespective of the number of output classes was evaluated with 13 multiclass and 9 binary datasets. All the datasets were generated using oligonucleotide-based technology where RNA was hybridized using Affymetrix arrays HG-U95/Hu6800/HuGeneFL/Hu35K. The gene expression values of all the datasets were computed using the Affymetrix GENECHIP MAS 4.0 analysis software. The data on small round blue cell tumors (SRBCTs), NCI60 (National Cancer Institute), and Lymphoma were acquired using a two-color cDNA platform with successive image analysis by means of the DeArray Software. To summarize, 22 datasets included in our experiments each have 2–11 distinct diagnostic categories, 24–253 samples (patients), and 182–54614 genes collected from different tissues under different experimental conditions. The number of samples per class is highly sparse and imbalanced (varies from 6 to 579).

**Table 1:** Characteristics of gene expression datasets used for analysis.

Dataset	#Genes	#Sam	#Cat	Source
**Multiclass**				
Acute Lymphoblastic Leukemia (ALL)	2526	248	6	Yeoh et al., 2002 [30]
Gastric Cancer (GC)	4522	30	3	Hippo et al., 2002[31]
National Cancer Institute NCI60 (NCI)	5244	61	8	Dudoit et al., 2002 [5]
Novartis (Nov)	1000	103	4	Su et al., 2002 [32]
Brain_Tumor (BT)	7129	42	5	Pomeroy et al., 2002 [33]
Glioblastoma(GB)	12625	50	4	Nutt et al., 2002 [11]
Leukemia (Leu)	5327	72	3	Armstrong et al., 2002 [34]
Endometrial Cancer (EC)	1771	42	4	Risinger et al., 2003 [35]
Childhood (Ch)	8280	60	4	Li et al., 2003 [36]
Bladder Carcinoma (BC)	1203	40	3	Dyrskjot et al., 2003 [37]
Global Cancer Map with repeated measurements (GCM_RM)	7129	123	11	Yeung et al., 2003 [38]
**Binary**				
Lung Cancer1 (Lun1)	10541	34	3	Dehan et al., 2007 [39]
Lung Cancer2 (Lun2)	12600	181	2	Gordon et al., 2002 [40]
Prostate Cancer (Pro)	12600	136	2	Singh et al., 2002 [41]
Ovary Cancer (Ova)	15154	253	2	Petricoin et al., 2002 [42]
Diffuse Large B-Cell Lymphoma (DLB)	5469	77	2	Shipp et al., 2002 [43]
Hypopharyngeal Cancer (Hypo)	9021	38	2	Cromer et al., 2004 [44]
Breast Cancer (Bre)	12625	24	2	Chang et al., 2005[45]
Breast / Colon Cancer (BCC)	182	104	2	Chowdary et al., 2006 [46]
Colorectal Carcinoma (CC)	2202	37	2	Laiho et al., 2007 [47]
Pancreatic Cancer (Pan)	54614	52	2	NCBI, 2009 [48]
Kidney Carcinoma (KC)	7457	36	2	NCBI, 2009 [48]

#Genes: number of genes, #Sam: samples, #Cat: categories

### Proposed architecture for analyzing cancer gene expression data

**Fig 1:**
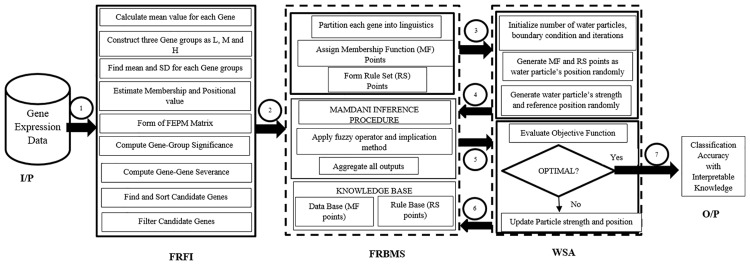
Architecture of the proposed FRFI-WSA approach for cancer gene expression data.

A clinical challenge concerning the limited number of patients (scarcity) that is skewed in favor of one group (disparity) with a huge number of genes (dimensionality) across many categories of cancer (multiclass) are the problems faced by clinicians during analysis of gene expression data for prediction of cancer [30–33]. To overcome these drawback, problem-specific computational techniques for multiclass cancer diagnosis was developed here. As shown in Fig 1, the implementation procedure of the proposed combinatorial approach can be viewed in seven phases. The first phase reads the input data into the FRFI method. It helps to find the candidate genes in the huge number of genes using well-narrated steps as presented in Fig 1. The candidate genes are then fed into FRBMS in the second phase to find the initial points for the membership function (MF) and rule set (RS). In the third phase, these initial MF points and RS are read into the WSA to generate a population of points as a water particle’s position. The generated points are submitted to the inference procedure of FRBS in the fourth phase to compute the correctly classified samples (Cs), the selected number of rules (Rs), and selected number of informative genes (Gs). The parameters Cs, Rs, and Gs calculated in the FRBMS are then input to the WSA in the fifth phase for evaluating the objective function, which determines optimality of the generated water particle’s position as a knowledge base. If the optimality criteria are not met, then the water particle’s strength and position are updated accordingly to generate a useful knowledge base which results in improved classification of samples. The fifth and sixth phases are repeatedly executed until the desired convergence criterion is achieved. In the final phase, acceptable classification accuracy with interpretable knowledge is generated in the form of if-then conditional statements that help to identify the cancer-causing genes. The details of subcomponents of the proposed architecture are given below.

### FRFI

Regardless of the dimensionality issue, the fuzzy rough set (FR) [49] effectively calculates the redundancy (severance) as well as relevance (significance) using f-information (FI) without discretizing the continuous gene expression values. The detailed concepts of the fuzzy set, rough set, fuzzy rough set and f-information is presented in S1 Appendix. Even though the FR offers a regimented means for FI-based gene selection, it becomes inadequate for the noisiness and poor dispersal of multiclass samples. Hence, it was upgraded with a fuzzy lower approximation [50] to compute FI extrinsically to filter a gene subset. Given an n × m matrix of gene expression data with “m” gene vectors, the goal of gene filtering is to produce an n × f gene expression data matrix with “f” filtered gene vectors, where f < m. The steps involved in computing FI using the FR are as follows.

1. Read the gene expression dataset *G*_*i*×*j*_ where I = 1, 2, … *m*; *c* and *j* = 1, 2, … *n*; *m* is the number of genes, *c* is a class label, and *n* is the number of samples.
2. Calculate the mean value *μ* = {*μ*_1_, *μ*_2_,…*μ*_*m*_, *μ*_*c*_} for each gene of all the samples and class labels.
3. Generate two gene groups (High H, Low L) by comparing each gene value with respective mean values, so that, H = {Genes with a value greater than its mean} and L = {Genes with a value lower than its mean}
4. Calculate the mean value of two gene groups for each gene, $\mu_{L}=\{\mu_{L_{1}},\mu_{L_{2,}},\ldots \mu_{L_{c}}\}$ and $\mu_{H}=\{\mu_{H_{1}},\mu_{H_{2,}},\ldots \mu_{H_{c}}\}$
5. The mean value calculated at step (iii) is considered the medium mean value, $\mu_{M}=\mu_{M_{1}},\mu_{M_{2,}},\ldots \mu_{M_{c}}$
6. Calculate the standard deviation for each mean value {*μ*_*L*_, *μ*_*M*_, *μ*_*H*_}: $\sigma_{L}=\{\sigma_{L_{1}},\sigma_{L_{2}},\ldots \sigma_{L_{c}}\}$, $\sigma_{H}=\{\sigma_{H_{1}},\sigma_{H_{2}},\ldots \sigma_{H_{c}}\}$ and $\sigma_{M}=\{\sigma_{M_{1}},\sigma_{M_{2}},\ldots \sigma_{M_{c}}\}$.
7. Calculate the membership value in lower fuzzy approximation spaces for each gene *G*_*i*×*j*_,
  $$\pi_{L}(G_{i\times j},\mu_{L_{{}_{i}}},\sigma_{L_{i}})=\left\{ \begin{array}{l} 2{\left(1-||G_{i\times j}-\mu_{L_{i}}||\right)}^{2},\frac{\sigma_{L_{i}}}{2}\leq ||G_{i\times j}-\mu_{L_{i}}||\leq \sigma_{L_{i}}\\ 2{\left(1-||G_{i\times j}-\mu_{L_{i}}||\right)}^{2},0\leq ||G_{i\times j}-\mu_{L_{i}}||\leq \sigma_{L_{i}}\\ 0,otherwise \end{array}\right.$$
8. Calculate the positional values $\left(P_{L}^{G_{i\times j}},P_{M}^{G_{i\times j}},P_{H}^{G_{i\times j}}\right)$ for each gene:
  $$P_{L}^{G_{i\times j}}=\frac{\pi_{L_{i\times j}}}{\pi_{L_{i\times j}}+\pi_{M_{i\times j}}+\pi_{H_{i\times j}}}$$
9. Construct the fuzzy equivalence partition matrix (FEPM) FP_i_ = $\left[\begin{array}{l} P_{L}{}^{G_{i\times j}}\\ P_{M}{}^{G_{i\times j}}\\ P_{H}{}^{G_{i\times j}} \end{array}\right]$ for each gene
10. Suppose *G*_*i*×*j*_ represents a gene and *G*_*c*_ represents a class label. Then the Gene-Group significance value is calculated as
  $$\begin{array}{ll} & \;\left|\frac{1}{n}\sum_{j=1}^{n}\left(P_{L}{}^{G_{i\times j}}\cap P_{L}{}^{G_{c\times j}}\right)-\frac{1}{n^{2}}\sum_{j=1}^{n}P_{L}{}^{G_{i\times j}}\sum_{J=1}^{n}P_{L}{}^{G_{c\times j}}\right|\;+\\ F_{sig}\left(G_{i\times j,}G_{c}\right) & =\;\left|\frac{1}{n}\sum_{j=1}^{n}\left(P_{H}{}^{G_{i\times j}}\cap P_{H}{}^{G_{c\times j}}\right)-\frac{1}{n^{2}}\sum_{j=1}^{n}P_{H}{}^{G_{i\times j}}\sum_{J=1}^{n}P_{H}{}^{G_{c\times j}}\right|\;+\\ & \left|\frac{1}{n}\sum_{j=1}^{n}\left(P_{M}{}^{G_{i\times j}}\cap P_{M}{}^{G_{c\times j}}\right)-\frac{1}{n^{2}}\sum_{j=1}^{n}P_{M}{}^{G_{i\times j}}\sum_{J=1}^{n}P_{M}{}^{G_{c\times j}}\right| \end{array}$$
11. Now, Gene-Gene Severance between *F*_*sig*_ and the remaining genes *G*_*rem*_ is calculated as
  $$\begin{array}{ll} F_{sev}\left(F_{sig_{x\times j},}\;G_{rem_{x\times j}}\right) & =\left|\frac{1}{n}\sum_{j=1}^{n}\left(P_{L}{}^{l_{rel_{x\times j}}}\cap P_{L}{}^{l_{rem_{x\times j}}}\right)-\frac{1}{n^{2}}\sum_{j=1}^{n}P_{L}{}^{l_{rel_{x\times j}}\sum_{j=1}^{n}P_{L}{}^{l_{rem_{x\times j}}}}\right|\;+\\ & \;\left|\frac{1}{n}\sum_{j=1}^{n}\left(P_{H}{}^{l_{rel_{x\times j}}}\cap P_{H}{}^{l_{rem_{x\times j}}}\right)-\frac{1}{n^{2}}\sum_{j=1}^{n}P_{H}{}^{l_{rel_{x\times j}}\sum_{j=1}^{n}P_{H}{}^{l_{rem_{x\times j}}}}\right|\;+\\ & \left|\frac{1}{n}\sum_{j=1}^{n}\left(P_{M}{}^{l_{rel_{x\times j}}}\cap P_{M}{}^{l_{rem_{x\times j}}}\right)-\frac{1}{n^{2}}\sum_{j=1}^{n}P_{M}{}^{l_{rel_{x\times j}}\sum_{j=1}^{n}P_{M}{}^{l_{rem_{x\times j}}}}\right| \end{array}$$
12. Calculate the FI value for each gene *G*_*i*×*j*_ using the formula *FI* = min|*F*_*sig*_−*F*_*sev*_| and sort them in descending order of FI values for filtering.

It is expected that the proposed method of fuzzifying the criterion function of FI with a rough set can filter genes extrinsically in a way similar to human intervention into gene identification.

### FRBMS

**Fig 2:**
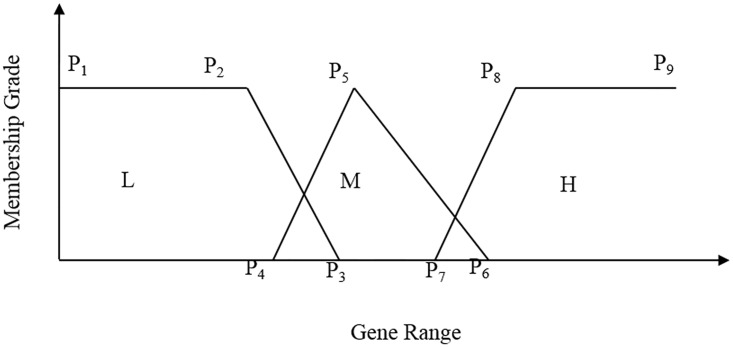
Partitioning of input genes in fuzzy space.

The filtered candidate genes from the FRFI method are partitioned into linguistics to generate the MF and RS points. As shown in Fig 2, this study includes three partitions such as low (“L”), medium (“M”), and high (“H”), and thus nine membership points (P_1_, P_2_, P_3_, P_4_, P_5_, P_6_, P_7_, P_8_, and P_9_) are required to encode each candidate gene. P_1_ and P_9_ are permanent to designate the limits of the gene expression value. The optimal values for other points are selected between the limits [P_1_, P_9_] for P_2_, [P_2_, P_9_] for P_3_, [P_2_, P_3_] for P_4_, [P_4_, P_9_] for P_5_, [P_5_, P_9_] for P_6_, [P_5_, P_6_] for P_7_, and [P_7_, P_9_] for P_8_. These points take floating-point numbers in which triplets P_1_, P_2_, P_3_ and P_7_, P_8_, P_9_ draw a trapezoidal MF and the triplet P_4_, P_5_, P_6_ draws a triangular MF.

**Fig 3:**
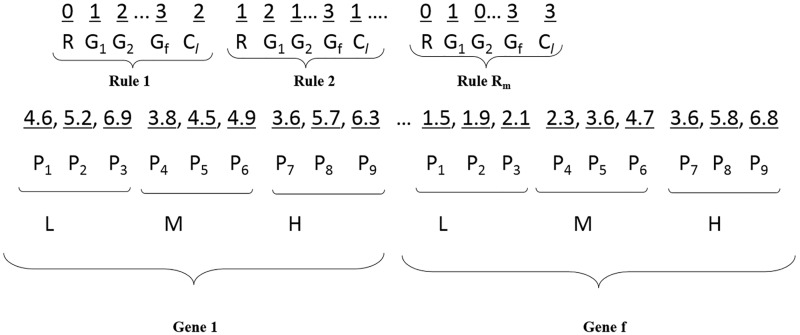
Representation of typical membership function (MF) points and rule set (RS) for FRBMS.

The representation of typical MF points and RS for FRBMS is shown in Fig 3. A rule choses integer numbers in three sections viz., Rule selection, Antecedent, and Consequent. “R” denotes a rule selection that can be either 0 or 1 to select or deselect the rule. G_1_, G_2_, G_3_ … G_f_ in the antecedent part represents filtered genes, denoting a random integer value among 0, 1, 2, and 3 to perform linguistic as well as gene selection. The consequent C_*l*_ takes any value among 0, 1, 2 … n to assign the category of cancer. These single MF and RS points are fed to WSA to initialize more MF and RS points randomly as a position for the initial water particle. Based on the procedural evaluation of WSA, a knowledge base is constructed that contains the optimal data base (MF points) and rule base (RS points). This knowledge base extracted by WSA is used in a Mamdani inference procedure to perform classification of samples.

### WSA

This is a new optimization algorithm [51, 52] inspired by the way water finds a drain in a sink. The learning principle of WSA is used to make the FRBMS as self-learning system by providing the knowledge base in the form of optimal MF and RS points. The WSA starts by initializing the control parameters like the number of water particles, boundary conditions, and iteration followed by random initialization of the position for water particles using the initial MF and RS points received from the FRBMS. Then, for each water particle position, WSA generates water particle’s strength and a reference position randomly. After that, each water particle’s position (i.e., MF and RS points) are evaluated using the objective function given in this equation:

$$MinimizeObj\;=\left(T_{s}-C_{s}\right)\;+\;\left(k_{1}\;\times \;R_{s}\right)+\;\left(k_{2\;}\times \;G_{s}\right)$$ (1)

where *T*_*s*_ is the total number of samples, *C*_*s*_ is the number of correctly classified samples, *R*_*s*_ is the selected rules from the maximum rules *R*_*m*_, and *G*_*s*_ is the selected number of genes from the filtered genes. *k*_*1*_ and *k*_*2*_ are constants used to amplify *R*_*s*_ and *G*_*s*_. The component (*T*_*s*_−*C*_*s*_) calculates error. The WSA approach used in this study attempts to minimize the error component and to improve accuracy of the system. Similarly, the component (*k*_*1*_ × *R*_*s*_) tries to produce a RS whose interpretability is addressed suitably by WSA. The component (*k*_*2*_ × *G*_*s*_) attempts to find out the minimal number of potential genes on the basis of the linguistic selection.

The optimality of the generated MF and RS points is checked during every iteration to yield the result. If the optimal points are not obtained, then the MF and RS points are updated iteratively using the strength and position update eqs (2) and (3):

$$\alpha_{new}=\alpha_{old}+\alpha_{q,ref}\times \left(x_{prevBest}-x_{q,ref}\right)+\alpha_{q,ref}\times \left(x_{gBest}-x_{p,old}\right)$$ (2)

$$x_{p,new}=\alpha_{new}+\alpha_{q,ref}\times \left(x_{p,old}-x_{q,ref}\right)$$ (3)

where *α*, *x*_*p*_, and *x*_*q*,*ref*_ are all randomly generated using the range given for the solution variable; *α*_*q*,*ref*_ is a random number generated between 0 and 1; *α*_*old*_ and *α*_*new*_ are the strength vectors of water particles during i^th^ and (i + 1)^th^ iterations. Similarly, *x*_*p*,*old*_ and *x*_*p*,*new*_ are the positions of water particles during i^th^ and (i + 1)^th^ iterations; *x*_*q*,*ref*_, *x*_*prevBest*_, and *x*_*gBest*_ denote the reference position, previous best position, and global best position of the water particle, respectively.

## Results

### FRFI-WSA for the global cancer map with repeated measurements (GCM_RM) dataset

The steps of the proposed FRFI-WSA are demonstrated for tumor data categories of the GCM_RM dataset, which contains 123 samples. Out of 123 samples, 96 and 27 are used for training and testing, respectively. Furthermore, this dataset has 11 categories of tumors with 7129 genes. The 96 training samples include all categories of tumors. Nonetheless, the set of 27 test samples does not include samples of breast, melanoma, and pancreatic tumors. Hence, in this simulation, both the training and testing samples are mixed to have a reasonable sample for each category. Similar consideration is given to other kinds of datasets. The distributions of classes among the training (#Tr) and the testing (#Te) samples of GCM_RM are given in Table 2.

**Table 2:** Distribution of the training and testing tumor data categories in the GCM_RM dataset.

Tumor Category	Total No. of Samples	Actual		Considered	
		#Tr	#Te	#Tr	#Te
Breast	7	7	0	4	3
Lung	6	4	2	4	2
Colorectal	10	7	3	7	3
Lymphoma	19	14	5	14	5
Melanoma	5	5	0	3	2
Uterus	9	7	2	7	2
Leukemia	29	23	6	23	6
Renal	8	5	3	5	3
Pancreas	7	7	0	4	3
Mesothelioma	11	8	3	8	3
CNS	12	9	3	9	3
Overall Total	123	96	27	88	35

**#**Tr: training data, #Te: testing data

At the first level of gene filtering, all the 123 samples are considered for the GRM dataset and other datasets as well. Initially, a fuzzy equivalence class (FEC) was calculated for an individual gene via the steps (i) through (viii) of FRFI. The FEC calculated for the individual gene is then used to produce an FEPM using step (ix) of FRFI. The FEC and FEPM calculated for the gene of GCM_RM whose accession id is AB002380_at are given in Table 3. Then Gene-Group significance is analyzed using step (x). Based on the Gene-Group significance value, genes are rated, and the gene with the highest significance value is designated as the first gene. Gene AB002380_at of GCM_RM has the highest significance value of 0.6489 and it is nominated as the top-rated significant gene. After significance calculation, Gene-Gene severance (redundancy) is analyzed among gene “AB002380_at” and the residual genes of the GCM_RM using step (xi) of FRFI as specified in Table 4.

**Table 3:** FEC and FEPM values for gene AB002380_at of the GCM_RM dataset.

**Fuzzy Equivalence Class for gene AB002380_at**					
FEC	S_1_	S_2_	…	S_122_	S_123_
Low	0.1578	0.2536	…	0.1925	0.4265
Medium	0.5269	0.6321	…	0.5262	0.5241
High	0.9417	0.9259	…	0.4534	0.9321
**Fuzzy Equivalence Partition Matrix for gene AB002380_at**					
FEPM	S_1_	S_2_	…	S_122_	S_123_
Low	0.1427	0.1426	…	0.1324	0.1758
Medium	0.7242	0.6321	…	0.5815	0.6519
High	0.9838	0.9162	…	0.9647	0.9235

S1….…S_123_: samples

**Table 4:** Gene group significance and gene-gene severance values of the GCM_RM dataset.

Gene No.	Gene ID	G_sig_	G_sev_
G_1_	A28102_at	0.193452	0.234561
G_2_	AB000114_at	0.152567	0.343587
G_3_	AB000115_at	0.124561	0.561924
…	…	…	…
G_7128_	Z97054_xpt2_at	0.156722	0.145623
G_7129_	Z97074_at	0.112345	0.532419

G_sig_: Gene significance, G_sev_: Gene severance

**Fig 4:**
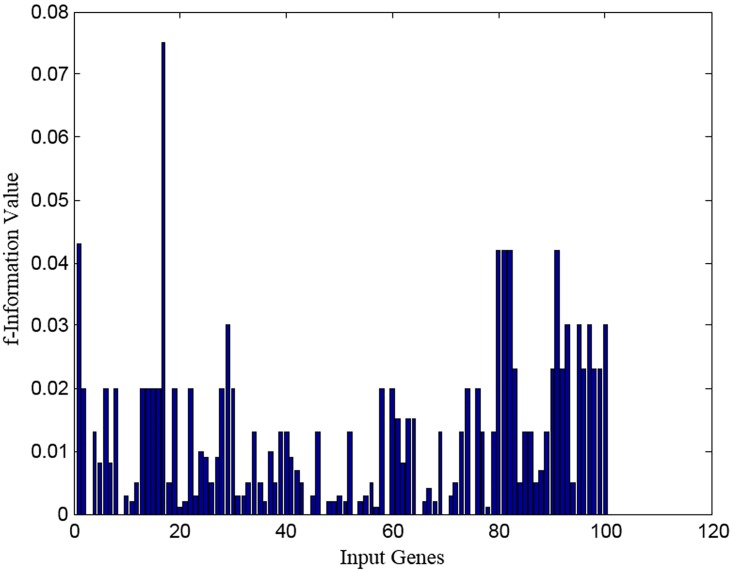
The F-information (FI) values of first hundred genes for GCM_RM dataset.

From the significance and severance values, an FI value for each gene is calculated using step (xii) of FRFI so that it maximizes the significance and minimizes severity. The FI values of first 100 genes are shown in Fig 4. There are variations among the FI values computed for each gene. The genes are arranged in descending order of FI values to filter out the top 50 genes from 7129 genes to achieve a good trade-off between significance and severance for further evaluation. Identification of the most significant gene among the initially filtered 50 genes is carried out using WSA, which aims to generate minimum rules with less informative genes to classify more samples by means of the FRBMS during classification.

Each rule is found to take 52 varying integer numbers (1 for *R*, 50 for “G_1_, G_2_, G_3_ … G_f_,” 1 for C_*l*_) as per the representation strategy given in Fig 3. The maximal number R_m_ of initial rules in the RS is determined heuristically by multiplying the number of classes (#Cat) in the dataset by 3 with the goal of obtaining at least a single rule for each category of cancer. For the GCM_RM dataset, 33 rules (11 × 3) are randomly initialized in the RS. Hence, the RS of GCM_RM contains 1716 integer numbers (33 × 52). Seven points are required to figure out the linguistic variables of every gene, and hence 350 floating-point numbers (7 × 50) are needed. The count of an integer variable differs from dataset to dataset depending on the number of cancer categories, whereas the count of a floating-point number is common for all the datasets.

The size of the initial solution space is considered within 20 to 50. Each position of the water particle is evaluated using the objective function (1) by changing the iterations from 10 to 100. The value for constants *k*_1_ and *k*_2_ in eq (1) is varied from two to five depending on the R_s_ and G_s_ obtained during a particular iteration. A maximum of 40 independent trials of experiments have been conducted by varying the water space as well as the iteration. The resulting performance of every particle inside water is examined. The finest results for GCM_RM datasets for 30 water spaces between 80 to 100 iterations were observed. A similar experiment was conducted for all other datasets used in this study. The selection of the most significant 16 genes in the RS along with their descriptions for identification of tumor categories among the 50 filtered genes are presented in S1 Table. The rule set gleaned for the GCM_RM dataset is presented in Table 5. Twenty-six rules were generated to achieve classification accuracy of 96.45%.

**Table 5:** The rule set generated for the GCM_RM dataset by the FRFI-WSA method.

Rule No.	Rule Set
R1	If (PDCD1 & OGDH) are low and MG81 is medium, then it is Breast cancer.
R2	If (PRMT1 & LGALS9) are medium and (X03453 & RAD51) are high, then it is Breast cancer.
R3	If (GLO1 & SLC25A13) are high and PRKAR1A is low, then it is Breast cancer.
R4	If J04423 is high, and GLO1 is low, and NCOR2 is high, then it is Lung cancer.
R5	If (RYR1 & SLC25A13) are low and (RAD51 & PRKAR1A) are medium, then it is Lung cancer.
R6	If NOP14-AS1 is high and (J04423 & NCOR2) are medium, then it is Colorectal cancer.
R7	If (PDCD1 & OGDH) are medium and MG81 is low, then it is Colorectal cancer.
R8	If J04423 is low and RBM42 is high, then it is Lymphoma.
R9	If PRMT1 is high and, (X03453 & LGALS9) are low, then it is Lymphoma.
R10	If (RYR1 & RBM42) are medium and PDCD1 is high, then it is Melanoma.
R11	If (GLO1 & PRKAR1A) are medium and PDCD1 is low, then it is Melanoma.
R12	If NOP14-AS1 is low and (J04423 & M24537B) are high, then it is Uterine cancer.
R13	If (PRMT1 & LGALS9) are high and (X03453 & RAD51) are medium, then it is Uterine cancer.
R14	If J04423 is medium and GLO1 is high and MG81 is low, then it is Uterine cancer.
R15	If NOP14-AS1 is high and M24537B is medium, then it is Leukemia.
R16	If (PDCD1 & OGDH) are medium and NCOR2 is low, then it is Leukemia.
R17	If PRMT1 is medium and (X03453 & LGALS9) are low, then it is Renal cancer.
R18	If J04423 is high, and RBM42 is medium and OGDH is low, then it is Renal cancer.
R19	If (RYR1 & SLC25A13) are medium and (RAD51 & PRKAR1A) are high, then it is Renal cancer.
R20	If (PRMT1 & NCOR2) are low and NOP14-AS1 is medium, then it is Pancreatic cancer.
R21	If X03453 is medium and (RBM42 & M24537B) are low, then it is Pancreatic cancer.
R22	If RYR1 is low and (MG81 & PDCD1) are high, then it is Pancreatic cancer.
R23	If PRMT1 is low and X03453 is high, and LGALS9 is medium, then it is Mesothelioma.
R24	If PRMT1 is low and (X03453 & LGALS9) are high, then it is Mesothelioma.
R25	If (PRKAR1A & SLC25A13) are high and (NOP14-AS1 & RAD51) are low, then it is CNS cancer.
R26	If (J04423 & M24537B) are low and (RYR1 & OGDH) are high, then it is CNS cancer.

In Table 6, the accession ID of the most significant genes is presented along with the selected linguistic label and tumor category, which help to identify the genes causing the tumor. Furthermore, the GCM_RM dataset was examined with a different number of initial rules such as four, five, and six. It ultimately resulted in 44, 55, and 66 rules in the RS. The selected optimum genes involved in a different RS are not distributed reasonably among common genes. Hence, it is understandable that the various subsets of genes are selected for categorizing the classes of patients. Nevertheless, the genes selected beyond 20 to 100 in the RS yielded a minor improvement (roughly 0.6%) in the classification. Hence, it could be said that the proposed approach shows robust performance with 26 generated rules because it utilizes 16 selected genes to classify 119 out of 123 samples in the GCM_RM dataset.

**Table 6:** Identification of the most significant genes and their linguistic label in the rule set for the classification of tumor categories for the GCM_RM dataset by FRFI-WSA.

Gene Name	Linguistic Label		
	Low	Medium	High
RBM42	Pancreas	Melanoma/Renal	Lymphoma
SLC25A13	Lung	Renal	Breast/CNS
J04423	CNS	Colorectal	Uterus
X03453	Lymphoma/Renal	Uterus/Pancreas	Breast/Mesothelioma
NOP14-AS1	CNS/Uterus	Pancreas	Colorectal/Leukemia
M24537B	CNS/Pancreas	Leukemia	Uterus
OGDH	Breast/ Renal	Colorectal/Leukemia	CNS
GLO1	Lung	Melanoma	Breast/Uterus
RAD51	CNS	Lung/Uterus	Breast/Renal
NCOR2	Pancreas/Leukemia	Colorectal	Lung
PDCD1	Breast/Melanoma	Colorectal/Leukemia	Melanoma/Pancreas
PRMT1	Mesothelioma/Pancreas	Breast/Renal	Lymphoma/Uterus
LGALS9	Lymphoma/ Renal	Breast/Mesothelioma	Uterus/ Mesothelioma
PRKAR1A	Breast	Lung/Melanoma	Renal/CNS
RYR1	Lung/Pancreas	Melanoma/Renal	CNS
MG81	Colorectal/Uterus	Breast	Pancreas

### Empirical results

#### Performance comparison and evaluation metrics

**Fig 5:**
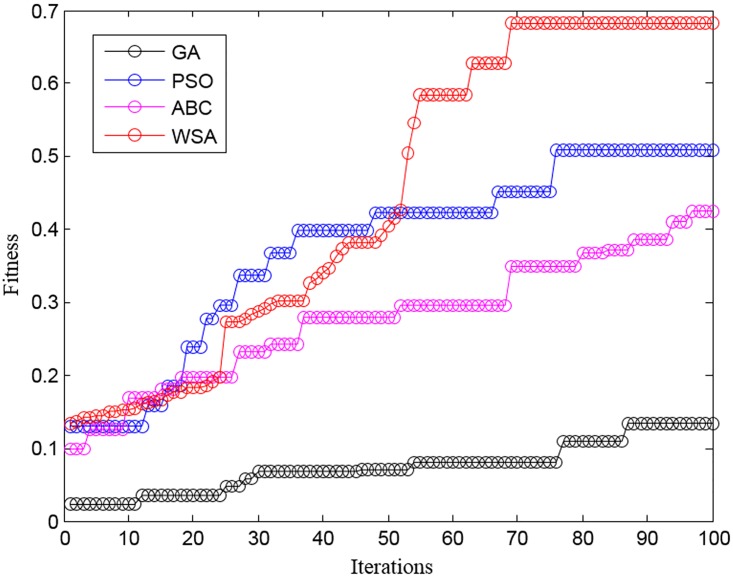
Convergence comparison of WSA with other methods for GCM_RM dataset.

The performance of the proposed WSA approach was compared with the competing methods such as GA [20], PSO [21], and ABC [22] on all the datasets. A comparison in convergence between the proposed WSA for the GCM_RM dataset and other approaches is shown in Fig 5. It is noteworthy that the convergence of other approaches is worse than that of the proposed WSA approach. Although the other approaches based on GA, PSO, and ABC are relatively good at tuning the MF, they consume more generations to converge. It is clear in the figure that both ABC and WSA show an abrupt rise in the fitness value whereas the GA and PSO approaches showed only a steady increase in the fitness value. The reason could be the more tunable parameters.

In Table 7, a comparison is presented between the proposed WSA and the other methods for all datasets. For each dataset (DS), the table shows the classification accuracy (CA%), number of genes (#Gs), and central processing unit (CPU) time (CT). All methods are credibly good in their performance, but it appears that PSO is a little faster than the others except WSA because of PSO’s simplified operations. Nonetheless, PSO did not produce an optimal solution better than ABC did. Even though ABC is relatively good at producing interpretable rules, it consumes more CPU time due to the different phases of bee operations in generating simple rules. In contrast, the proposed WSA acquired a quick desired fitness value with a minimum number of most significant genes for all the binary and multiclass datasets used in this study. It is indicated that the properly tuned regularization parameters by optimization using WSA can be possible to extend the proposed approach to classify binary and multiclass samples for cancer gene expression datasets.

**Table 7:** Comparison of the performance of the water swirl algorithm with existing methods on all datasets.

DS	#Gs				CA%				CTs			
	GA	PSO	ABC	WSA	GA	PSO	ABC	WSA	GA	PSO	ABC	WSA
ALL	41	38	35	23	84.23	88.42	92.78	95.12	321.45	298.43	194.65	173.42
GC	38	34	33	26	86.87	92.41	94.76	95.23	312.45	284.87	223.45	165.98
NCI	40	39	29	22	84.69	87.45	91.28	96.89	292.43	290.14	264.52	187.56
Nov	42	37	32	24	85.89	87.43	91.67	95.64	296.31	278.23	250.42	176.43
BT	34	34	28	19	87.25	90.45	89.45	95.12	218.46	188.35	158.25	121.49
GB	36	33	36	26	83.96	87.45	91.23	96.42	267.35	243.76	186.12	156.81
Leu	32	29	27	24	84.56	87.56	90.58	96.79	291.43	258.43	192.23	157.56
EC	39	37	36	24	84.12	86.49	92.56	96.12	246.71	217.38	183.46	162.53
Ch	38	37	30	23	86.45	82.45	92.47	95.12	245.83	221.64	193.46	153.29
BC	42	38	33	27	90.15	89.61	92.49	94.19	257.14	243.87	225.32	196.78
GCM_RM	32	29	26	18	84.59	85.67	90.56	96.45	294.12	256.45	198.25	165.54
Lun1	45	42	34	29	91.32	93.23	90.46	94.71	258.98	247.32	194.85	153.59
Lun2	38	36	35	32	83.48	85.29	90.59	96.87	238.14	195.42	168.12	148.12
Pro	25	22	19	12	84.52	86.47	90.32	95.73	262.14	205.46	171.31	124.12
Ova	26	21	18	12	87.29	94.36	95.54	98.56	258.69	201.13	187.89	143.65
DLB	21	19	17	14	84.78	92.57	94.87	97.45	294.78	268.59	237.56	165.87
Hypo	39	35	31	27	85.43	81.26	91.49	98.23	295.67	287.45	163.67	151.25
Bre	38	35	29	23	83.25	88.49	93.21	96.46	284.35	256.45	218.34	187.19
BCC	27	29	24	21	84.58	88.19	93.45	98.76	275.34	263.46	246.12	223.14
CC	25	21	17	14	86.43	89.12	93.14	96.34	275.87	251.23	231.98	201.49
Pan	15	12	10	7	89.49	94.26	95.12	98.29	247.36	203.62	168.23	114.29
KC	28	24	19	16	85.48	91.26	95.45	96.82	283.28	271.54	236.42	178.56

DS: dataset, #GS: number of genes, CA: classification accuracy, CT: central processing unit time

#### The Monte-Carlo cross-validation (MCCV) method

**Fig 6:**
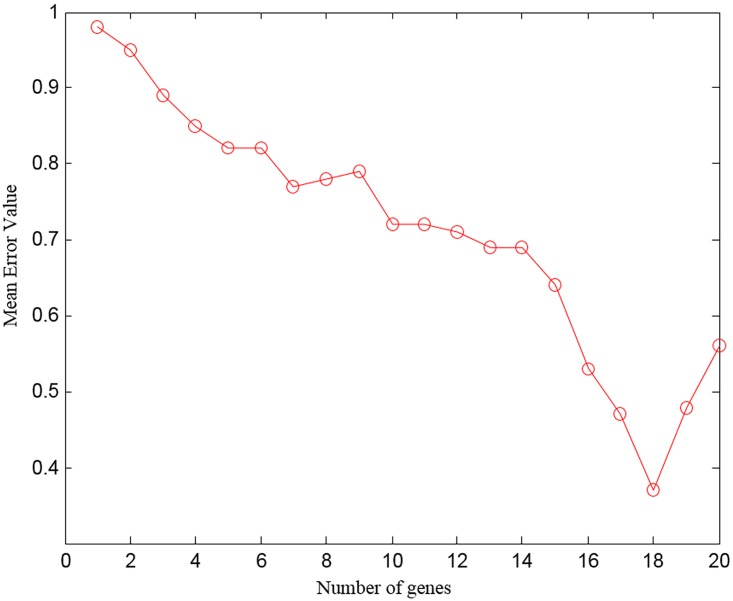
Generalization ability of WSA for GCM_RM dataset.

The performance of the proposed approach in terms of generalization was assessed using MCCV [53, 54] method. The mean value of the error calculated for the GCM_RM dataset using MCCV is presented in Fig 6. One can see that the error rate diminishes as the number of genes rises at every trial. Nevertheless, beyond 16 genes, the error rate surges to some extent. Hence, it is clear that a reasonably limited set of genes is sufficient to categorize the diverse cancer classes competently. Thus, the proposed FRFI-WSA approach can identify meaningful genes that cause cancer effectively with great precision for the classification of 11 tumor categories in GCM_RM datasets. Similar generalization performance was observed in all other datasets used in this study.

#### Wilcoxon’s signed-rank test

To evaluate noteworthy dissimilarities in outcomes between the competing methods and the proposed approach, Wilcoxon’s signed-rank test [21] was used. Table 8 presents the effects of the proposed approach are compared with those of the other methods for gene selection and knowledge acquisition. In this table, “r+” denotes the number of times the first method is superior to the second, and “r-“means the grades for disagreeing with the result. The null hypothesis “h” related to the Wilcoxon’s test is rejected (rej) because ρ < α = 0.01 in all comparisons favor WSA owing to variance in r+ and r- values. The results indicate that the fuzzy lower approximation space for computing significance and severance values of genes can deliver improvements in all metrics better than the existing methods can.

**Table 8:** Comparison of the performance of the water swirl algorithm with existing methods by Wilcoxon’s signed rank test on all datasets.

Comparison	GA Vs WSA				PSO Vs WSA				ABC Vs WSA			
	*r*^*+*^	*r*^*-*^	*ρ*	*h*	*r*^*+*^	*r*^*-*^	*ρ*	*h*	*r*^*+*^	*r*^*-*^	*ρ*	*h*
No. of Rules	5	86	0.53	*rej*	6	61	0.62	*rej*	3	62	0.31	*rej*
No. of Genes	6	78	0.45	*rej*	7	86	0.53	*rej*	5	65	0.51	*rej*
Accuracy	2	51	0.56	*rej*	4	48	0.82	*rej*	6	67	0.75	*rej*
Interpretability	6	75	0.91	*rej*	12	56	0.63	*rej*	11	69	0.79	*rej*
CPU Time	8	71	0.56	*rej*	18	38	0.51	*rej*	13	56	0.76	*rej*

#### The receiver operating characteristics (ROC) curve

**Fig 7:**
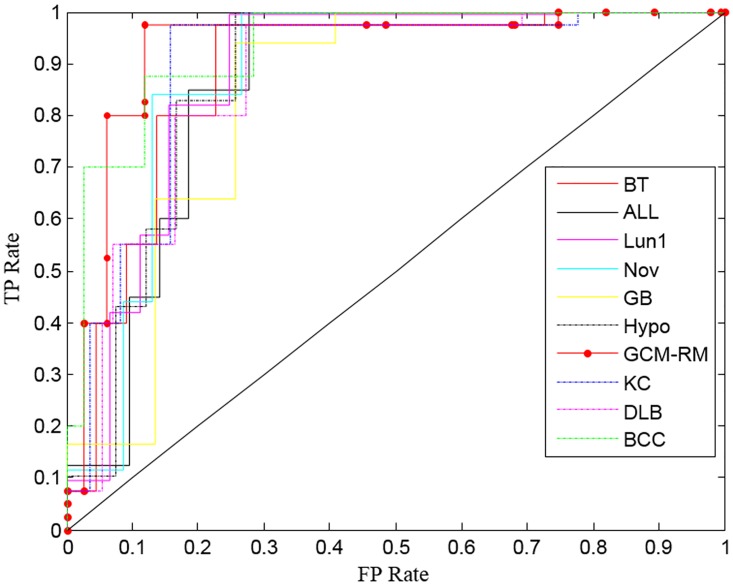
Receiver operating characteristics curve analysis for selected datasets by FRFI-WSA.

The ROC curve was drawn to understand the strength of the proposed FRFI-WSA using the true positive rate (TPR) against the false positive rate (FPR) in diverse cut points (Fig 7) [21, 55]. The proposed approach shows the ROC curve nearer to the higher left corner for all the data sets (for clear visualization, ROC curves are shown only for selected datasets). Our proposed approach has shown the highest sensitivity and specificity for all the datasets except for SRB and Car. Even though the proposed approach yields a lower value of the area under the curve (AUC) for SRB and Car datasets, this shortcoming does not disqualify the proposed approach as a screening test for cancer diagnosis because the effect of this shortcoming on performance is negligible.

#### Interpretability and gene ontology analysis

Readability and comprehensibility [56] are the two key valuation metrics to assess the interpretability of rules. The former deals with the model description that is quantified using the indices like coverage of the rules (*R*_*cov*_), accuracy of the rules (*R*_*acc*_), goodness of the rules (*R*_*gud*_), average rule length (*A*_*rl*_), average fired rules (*A*_*fr*_), and average confidence firing degree of the rules (*A*_*cfd*_). Values of those indices for every generated rule/RS can be obtained using eqs (4 to 9).

$$R_{\text{cov}}=\frac{N_{con}}{\#S}$$ (4)

$$R_{acc}=\frac{N_{pro}}{N_{con}}$$ (5)

$$R_{gud}=\frac{PCS_{fd}-NCS_{fd}}{TCS_{fd}}$$ (6)

$$A_{rl}=\frac{T_{rl}}{\#R}$$ (7)

$$A_{fr}=\frac{T_{fr}}{\#R}$$ (8)

$$A_{cfd}=\frac{A_{fd}}{\#S}$$ (9)

where *N*_*con*_ is the count of samples concealed by rule *R* in the total number of samples *#S*, and *N*_*pro*_ is the count of samples properly classified by *R* in *N*_*con*_. *PCS*_*fd*_, *NCS*_*fd*_, and *TCS*_*fd*_ are the firing degrees of positive, negative, and total covered samples, respectively. *T*_*rl*_ is the total rule length, i.e., the count of linguistic variables, *T*_*fr*_ is the total number fired rules, *A*_*fd*_ is the average firing degree of a rule, and *#R* is the total number of rules. The values of the indices for all the datasets are reported in Table 9. Throughout the execution, the proposed WSA tunes the MF points of each gene so that there is a reasonable overlap among the curves of linguistics. WSA also tries to ignore the MF points that attempt to go out of range. Likewise, the semantic label gained for each gene results in a reasonable length for each rule to use it compactly. The linguistic values (low, medium, and high) associated with each gene can help a physician to identify the patient’s distinct genomic contour to produce a verdict. The confidence about the average firing degree shows that the rules produced by WSA are fired more recurrently and have a tendency to be cofired with other rules. To avoid redundancy and to improve the compactness and interpretability without losing the classification accuracy, the rules with the lowest firing degrees are not included in this study.

**Table 9:** Reliability analysis of the rule set generated by FRFI-WSA in all datasets.

*DS*	*#R*	*R*_*cov*_	*R*_*acc*_	*R*_*gud*_	*A*_*rl*_	*A*_*fr*_	*A*_*cfd*_
ALL	22	15.87	85.46	0.182	5.31	10.19	0.432
GC	31	14.98	87.43	0.453	9.76	7.46	0.652
NCI	19	12.40	82.37	0.517	7.22	9.37	0.598
Nov	18	13.69	82.34	0.431	7.23	10.15	0.532
BT	11	11.02	84.75	0.795	5.91	8.34	0.567
GB	15	11.78	84.19	0.639	8.41	9.57	0.591
Leu	8	14.49	82.09	0.765	6.98	8.35	0.687
EC	9	15.76	86.34	0.653	9.54	9.16	0.639
Ch	9	14.67	86.71	0.652	8.56	10.21	0.546
BC	11	11.78	89.32	0.693	9.21	9.45	0.586
GCM_RM	26	12.69	83.39	0.823	6.92	9.95	0.535
Lun1	23	15.87	84.99	0.754	8.46	8.42	0.462
Lun2	7	10.40	80.68	0.546	7.70	8.49	0.536
Pro	5	11.76	82.09	0.576	7.20	9.46	0.621
Ova	9	16.82	84.62	0.679	5.23	9.20	0.503
DLB	8	18.47	84.16	0.959	5.60	8.06	0.530
Hypo	11	16.45	85.12	0.643	7.34	9.12	0.513
Bre	12	16.18	83.23	0.798	6.45	9.82	0.653
BCC	9	16.23	86.54	0.475	8.67	10.23	0.543
CC	10	18.41	88.45	0.467	6.813	9.14	0.614
Pan	9	10.48	83.67	0.562	5.05	9.48	0.634
KC	7	11.44	84.37	0.498	7.64	8.45	0.597

DS: datasets, #R: rules, *R*_*cov*_: coverage of the rules, *R*_*acc*_: accuracy of the rules, *R*_*gud*_: goodness of the rules, *A*_*rl*_: average rule length, *A*_*fr*_: average fired rules, *A*_*cfd*_: average confidence firing degree of the rules

Comprehensibility of the rules (which deals with explanation of the system concerning the inference complexity of rules) is analyzed using the information on cofiring of rules. For each rule *R*, the number of instances fired individually (*IF*) and simultaneously (*SF*) with every neighboring rule are recorded to compute a cofiring measure, *CF*, using the following equation:

$$CF_{ij}\;=\;\{\begin{array}{c} \frac{SF_{ij}}{\sqrt{IF_{i}\;.\;}IF_{j}}\;,\;if\;i\;\neq \;j\\ 0\;,\;if\;i\;=\;j \end{array}$$ (10)

Then the number of premises *P* in each rule is counted for computing the comprehensibility index (*CI*) using this equation:

$$CI=\;\sum_{i=1}^{r}\sum_{j=1}^{r}\left[\left(P_{i}\;+\;P_{j}\right)\;.\;CF_{ij}\right]$$ (11)

Where *r* is the total number of rules. Based on a heuristic threshold (*T*) between 0 and 1, the cofiring comprehensibility index (*CFCI*) is computed using eq (12) to understand the implied and clear semantics set in the fuzzy partitions and reasoning as well.

$$CFCI\;=\;\{\begin{array}{c} 1\;-\;\sqrt{\frac{CI}{T}}\;if\;CI\leq \;T\\ 0,\;otherwise \end{array}$$ (12)

**Fig 8:**
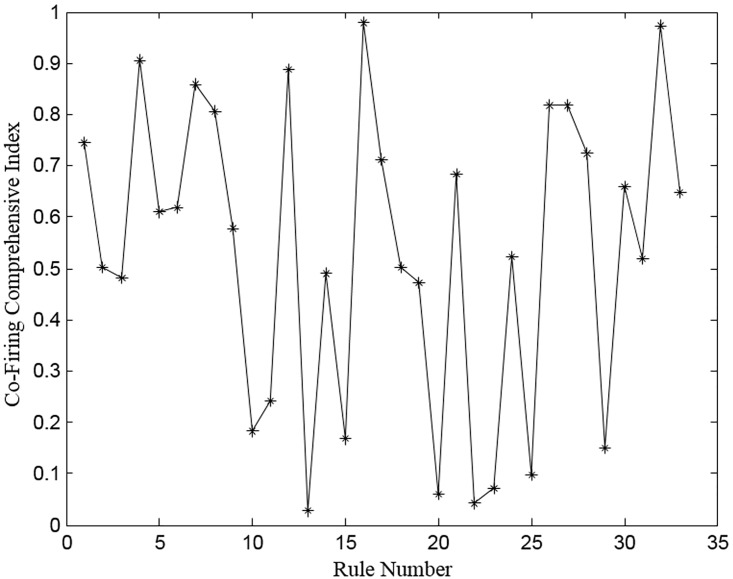
Comprehensibility of the generated rules by WSA for GCM_RM dataset.

The details of such analysis are illustrated in Fig 8 for the rules of the GCM_RM dataset. All the rules generated by WSA without any rule selection were used for examining its comprehensibility. Rule R_16_ has the largest *CFCI* value, while rule R_13_ has the smallest value. We found that the majority of the samples are fired between regions R_1_ and R_9_. Because R_16_ and R_32_ cover many problem instances, they overlap with the rules among R_1_ and R_9_. Linguistic simplification is carried out by combining rules R_26_ and R_27_ showing a similar *CFCI* value. As anticipated, it is easy to see that the evidence related to the new fused rules varies for FRBMS with the complete RS. Likewise, elimination of certain rules is done to fine-tune the system performance. We found that the accuracy of the system is improved after elimination of rules R_13_, R_20_, R_22_, R_23_, and R_25._ The interpretability analysis confirmed that the rules produced by the proposed WSA for all the datasets are transparent and comprehensible as well meet the requirements for understanding cancer gene expression data.

During gene expression-based cancer diagnosis, in addition to finding the subset of potential genes causing the cancer, the researcher is expected to trace out the physiognomies of the causative genes in terms of their part in multiple cancer classes [57]. The GO Sim package in the R platform [22] was used to compute the similarity value for the genes identified in the GCM_RM dataset using the GO terms. It is noteworthy that the genes are related to DNA metabolism and are enriched only in categories repair, positive regulation, reduction, cell size, development, and assembly. The nitrogen compound metabolic process of gene AFFX-CreX-3_st has an “is a” relation with GO:0006328 and is involved in a DNA metabolic process.

The primary metabolic process of AB000464_at has an “is a” relation with GO:004891, and the cellular process of AFFX-PheX-3_at has an “is a” relation with GO:006813. The process of cellular nitrogen compound metabolism relevant to Z49107_s_at has a “part of” relation with GO:000524 and GO:004271. The process of nucleobase-containing compound metabolism relevant to M33336_at has a “part of” relation with GO:013608 and GO:0044167. It was confirmed that the genes selected are involved in a DNA metabolic process, encode proteins associated with critical substances implicated in cancer. Such substances promote angiogenesis; help to elude apoptosis; increase differences from normal tissues; and enhance independent progression signs that lead to perfect prediction of cancer. Furthermore, the biological processes are consistent with the molecular activities that occur in active and proliferating cells of a cancer. The inequitable control of genes produced by the proposed procedure defines the extracellular environments that are important to understand the communications between the cells. Because most of the cancer genes restrained by the latest technology do not have entries in the GO database, it is not feasible to construct similarity relations between cancer genes for all the datasets used in this study. Overall, the refinement power of the nominated genes and their linguistics in the proposed model are sufficient to detect samples of a certain type of cancer and then to quickly rule out healthy samples.

## Discussion

In this study, we propose a new combined FRFI-WSA approach for designing an FRBMS to analyze gene expression data for cancer diagnosis. The WSA method showed the highest classification accuracy for detection of cancer genes in comparison with the GA, PSO, and ABC algorithms (Fig 5). Furthermore, the proposed approach showed the highest diagnostic sensitivity and specificity in the ROC analysis for estimation of classification performance. The superior performance of FRFI-WSA is obvious because the implementation of gene filtering in this study maximizes the gene-class relevance, minimizes the gene-gene redundancy, and arranges genes in an increasing order of the FI values without dependence on the classifier model. In addition, the most relevant genes associated with cancer were identified by the WSA, which attempts to optimize the RS and MF required for classification of samples using an FRBMS.

The combined FRFI-WSA approach quickly attained a desired fitness value using shorter computing time and a minimal number of rules for identification of the most significant cancer genes in comparison with the GA [20] and PSO [21] techniques (Table 7). This is probably because WSA is based on the novel strength and position update eqs (2) and (3), respectively, and simplifies operations with fewer or no parameters, thus rapidly extracting the RS and MFs. The fuzzy model integrated into GA reported in reference [19] deals only with binary data using a wide range of genes for classification of cancer genes. Moreover, it was also demonstrated that finding an optimal number of genes for multiclass problems is more beneficial for diagnosis of cancer. The ensemble combinatorial search is integrated into GA [14] as a single objective GA for optimization of the ensemble technique to classify class-imbalanced datasets. Nonetheless, a single objective GA attempts to locate solutions closer to the local optimum and hence the average error is much greater than in the proposed approach, which finds global optimal solutions for the classification. Hence, the proposed FRFI-WSA approach can effectively identify the most relevant genes associated with cancer (16 genes) with great precision (96.5%) and to generate understandable compact rules with fewer parameters for the classification of multiclass cancer categories. The classification performance of the FRFI-WSA according to cross-validation also proved that the two levels of gene selection implemented in this approach can eliminate or do not include some of the noisy genes that worsen classification performance.

The optimization using WSA in the present study effectively extracts comprehensible RS (26) and understandable linguistics for an MF for classifying the multiclass cancer samples. These data are also supported by another study [14], where the repeated tuning of an MF and RS was carried out by the optimization method could achieve the dimensionality challenges and multiple-class imbalanced data for optimal classifications. The lack of previous studies with the application of WSA for gene selection and RS based on multiclass gene expression datasets, making it difficult to compare our results directly is also one of the limitations in this study. Although the proposed model is better at identifying genes that are strongly responsible in order to classify different types of cancer, it consumes time, particularly in generating fuzzy equivalence class. In the future, the complexity of generating a fuzzy equivalence class by the FRFI method can be reduced by evaluating the Cartesian product using a fuzzy lower approximation for more rapid selection of a smaller subset of genes without any skewedness to multicategory data. However the proposed classifier model based on gene expression datasets extracted the most relevant genes associated with cancer by WSA method. Furthermore, the employment of other global optimization techniques such as genetic swarm and ant bee algorithms could be combined along with WSA method to generalize the interpretable rules with most relevant genes for cancer. In addition, further study also needed to verify the performance of the proposed approach to investigate the similarities of the gene expression data generated from other platforms such as Illumina, Agilent, etc. Our study revealed that the FR implemented here computes the FI without losing the biological meaning of the gene expression and should be helpful for identification of potential genes. Next, the WSA method will produce highly interpretable rules and will classify the maximal number of samples using an FRBMS better than the existing methods reported in the literature [14, 19–21]. Thus, the two levels of gene selection implemented in this study result in an efficient diagnostic system with lower complexity. Furthermore, the proposed approach reduces the computational cost and thus improves the classification accuracy of an FRBMS. In addition, the highest sensitivity and specificity in the selected multiclass datasets strongly indicate that the new FRFI-WSA approach is practically useful for construction of an effective system for making diagnostic decisions about cancer.

## Supporting Information

S1 Appendix The detailed concepts of the fuzzy set, rough set, fuzzy rough set and f-information. (PDF)

S1 Table Identification of the most significant genes along with their descriptions for the GCM_RM dataset by FRFI-WSA. (DOC)
