# Supplementary material for: Automated Detection of Cancer Associated Genes Using a Combined Fuzzy-Rough-Set-Based F-Information and Water Swirl Algorithm of Human Gene Expression Data
Source: PLoS One. 2016 Dec 9;11(12):e0167504. doi: 10.1371/journal.pone.0167504 (PMC5148587; doi:10.1371/journal.pone.0167504)
Supplement: S1 Appendix — (PDF) [file pone.0167504.s001.pdf]

Fuzzy set

Fuzzy set theory generalizes classical set theory to allow partial membership with a smooth boundary. The degree of membership in a set is expressed by a number between 0 and 1. 0 means entirely not in the set, 1 means completely in the set, and a number in between means partially in the set. Mathematically, a fuzzy set A in the universe of discourse X is defined to be a set of ordered pairs,

$$A = \{(x, \mu_A(x)) \mid x € X\} \qquad (A.1)$$

where $\mu_A(x)$ is called the membership function of x in A. Triangular and Trapezoidal are the most commonly used membership function.

Rough set

Rough Set theory handles uncertainty and vagueness present in a set of objects using two approximations, called lower and upper approximations. Given a finite set of objects $U = \{x_1, x_2, . . . , x_n\}$ described with a set of attributes $A = \{a_1, a_2, . . . , a_m\}$, each object $x_i \in U$ can be formulated as a vector $x_i = \{x_{i1}, x_{i2}, . . . , x_{im}\}$, where x_{ij} is the jth feature value of sample x_i . Let X be a subset of objects. The lower and upper approximations of X with respect to A are defined as $AX = \{x_i \in U|[x_i]A \subseteq X\}$ and $AX = \{xi \in U|[xi]A \cap X$ _= $\varnothing\}$, respectively. The lower approximation of X consists of the samples whose equivalence classes consistently belong to X, while its upper approximation is the subset of samples whose equivalence classes have objects in X.

Fuzzy rough set

Let $\langle U, R\rangle$ represent an approximation space, where *U* is a universe of discourse and *R* is an equivalence relation on *U*. Consider a subset *X* of *U*. *X* is called a rough set if it has a nonempty boundary set otherwise it is crisp. Selecting genes using rough set discretize their expression values and hence many researchers combined the fuzzy with rough set. Using fuzzy rough set, an object in *X* might be related to many fuzzy equivalence classes with a membership degree between [0, 1]. A fuzzy lower approximation and upper approximation can be stated using (1) and (2)

$$\mu_{\underline{X}}(F_i) = \sup_{F \in U/P} \min\{\mu_F(x), \inf_{y \in U} \max\{1 - \mu_F(y), \mu_X(y)\} \qquad (A.2)$$

$$\mu_{\overline{X}}(F_i)=\sup_{F\in U/P}\ \min\{\mu_F(x),\sup_{y\in U}\ \min\{1-\mu_F(y),\mu_X(y)\}\qquad \text{(A.3)}$$

Here F_i is fuzzy equivalence class (FEC), $\mu_F(x)$ is a membership degree of object x to fuzzy equivalence class F_i, P is a subset of attribute from set of attributes A.

***f*-Information**

f-information is a specific class of information measure that aims to calculate the distance between a given joint probability p_{ij} and the joint probability when the variables are independent $p_i p_j$. Some commonly used f-information measures are V-information, I_α – information, $M_\propto$ -information, and $X^\propto$ - information. Let $P_1 = \{p_i | i = 1,2,\ldots,n\}$, $P_2 = \{p_j | j = 1,2,\ldots,n\}$ and $P = \{p_{ij} | i = 1,2,\ldots,n; j = 1,2,\ldots,n\ \}$ represent two marginal probability distributions and their joint probability distributions respectively. The f-information measures are represented in the equations as,

V-information; $V(P||P_1 \times P_2) = \sum_{i,j}|p_{ij} - p_i p_j|$ (A.4)

$I_\propto$-Information ;$I_\propto(P\|P_1 \times P_2) = \frac{1}{\propto(\propto-1)}\sum_{i,j}\frac{(P_{ij})^\propto}{(P_i P_j)^{\propto-1}} - 1;\propto\neq 0;\propto\neq 1$ (A.5)

$M_\propto$-Information;$M_\propto(P\|P_1 \times P_2) = \sum_{ij}\left|(P_{ij})^\propto - (P_i P_j)^\propto\right|^{1/\propto}$;$0<\propto< 1$ (A.6)

$X^\propto$-Information; $X^\propto(P\|P_1 \times P_2) = \sum_{i,j}\frac{|P_{ij} - P_i P_j|^\propto}{(P_i P_j)^{\propto-1}}$;$\propto> 1$ (A.7)

All these measure divides the continuous expression values of a gene into discrete values followed by computing the marginal and the joint probability distributions. But this results inherent error and reduce the classification accuracy. It can be overcome by combining the concepts of fuzzy and rough set to define a new criterion function for *f*-information which effectively handle the real valued gene expression data.
