# Supplementary material for: Automated Detection of Cancer Associated Genes Using a Combined Fuzzy-Rough-Set-Based F-Information and Water Swirl Algorithm of Human Gene Expression Data
Source: PLoS One. 2016 Dec 9;11(12):e0167504. doi: 10.1371/journal.pone.0167504 (PMC5148587; doi:10.1371/journal.pone.0167504)
Supplement: S1 Table — (DOC) [file pone.0167504.s002.doc]

| **Probe ID** | **Gene Name** | **Gene Description** |
| --- | --- | --- |
| AC002115_rna2_at | RBM42 | RNA binding motif protein 42 |
| AC002450_at | SLC25A13 | solute carrier family 25 member 13 |
| AFFX-BioDn-3_st | J04423 | gene dethiobiotin synthetase |
| AFFX-CreX-3_st | X03453 | Bacteriophage P1 cre recombinase protein |
| AB000464_at | NOP14-AS1 | NOP14 antisense RNA 1 |
| AFFX-PheX-3_at | M24537B | subtilis pheB, pheA genes corresponding to nucleotides 2017-3334 |
| D10523_at | OGDH | oxoglutarate dehydrogenase |
| D13315_at | GLO1 | glyoxalase I |
| D14134_at | RAD51 | RAD51 recombinase |
| S83390_s_at | NCOR2 | nuclear receptor corepressor 2 |
| U64863_at | PDCD1 | programmed cell death 1 |
| Y10807_s_at | PRMT1 | protein arginine methyltransferase 1 |
| Z49107_s_at | LGALS9 | galectin 9 |
| M33336_at | PRKAR1A | protein kinase cAMP-dependent type I regulatory subunit alpha |
| J05200_rna1_s_at | RYR1 | ryanodine receptor 1 |
| HG909-HT909_at | MG81 | Protein |

**S1 Table. Identification of the most significant genes along with their descriptions for the GCM_RM dataset by FRFI-WSA**
